# The Gut Microbiota Composition in Dichorionic Triplet Sets Suggests a Role for Host Genetic Factors

**DOI:** 10.1371/journal.pone.0122561

**Published:** 2015-04-14

**Authors:** Kiera Murphy, Carol Anne O’ Shea, C. Anthony Ryan, Eugene M. Dempsey, Paul W. O' Toole, Catherine Stanton, R. Paul Ross

**Affiliations:** 1 Food Biosciences Department, Teagasc Food Research Centre, Fermoy, Co Cork, Ireland; 2 School of Microbiology, University College Cork, Cork, Ireland; 3 Department of Neonatology, Cork University Maternity Hospital, Cork, Ireland; 4 Alimentary Pharmabiotic Centre, University College Cork, Cork, Ireland; 5 Infant Centre, University College Cork, Cork, Ireland; 6 College of Science, Engineering and Food Science, University College Cork, Cork, Ireland; The University of Hong Kong, HONG KONG

## Abstract

Monozygotic and dizygotic twin studies investigating the relative roles of host genetics and environmental factors in shaping gut microbiota composition have produced conflicting results. In this study, we investigated the gut microbiota composition of a healthy dichorionic triplet set. The dichorionic triplet set contained a pair of monozygotic twins and a fraternal sibling, with similar pre- and post-natal environmental conditions including feeding regime. V4 16S rRNA and *rpoB* amplicon pyrosequencing was employed to investigate microbiota composition, and the species and strain diversity of the culturable bifidobacterial population was also examined. At month 1, the monozygotic pair shared a similar microbiota distinct to the fraternal sibling. By month 12 however, the profile was more uniform between the three infants. Principal coordinate analysis (PCoA) of the microbiota composition revealed strong clustering of the monozygotic pair at month 1 and a separation of the fraternal infant. At months 2 and 3 the phylogenetic distance between the monozygotic pair and the fraternal sibling has greatly reduced and by month 12 the monozygotic pair no longer clustered separately from the fraternal infant. Pulse field gel electrophoresis (PFGE) analysis of the bifidobacterial population revealed a lack of strain diversity, with identical strains identified in all three infants at month 1 and 12. The microbiota of two antibiotic-treated dichorionic triplet sets was also investigated. Not surprisingly, in both triplet sets early life antibiotic administration appeared to be a major determinant of microbiota composition at month 1, irrespective of zygosity. By month 12, early antibiotic administration appeared to no longer exert such a strong influence on gut microbiota composition. We hypothesize that initially host genetics play a significant role in the composition of an individual’s gut microbiota, unless an antibiotic intervention is given, but by month 12 environmental factors are the major determinant.

## Introduction

Microbial colonization of the infant gut is an essential process since microbiota-host interactions play a key role in host health. The gut microbiota have been shown to play important roles in the development and maturation of the immune system, metabolic pathways and in the bi-directional communication between the GI tract and the central nervous system (CNS), the so called gut-brain axis [1–5]. Early life perturbations in the microbiota can alter susceptibility to various gastrointestinal, immunological and neurological disorders [2, 4]

The idea that the foetus resides in a sterile environment in utero and that microbial colonization of the new-born begins at birth has been widely accepted. However, recent studies are challenging this with evidence for maternal microbial transmission to foetuses in utero in animals and reports of the placenta and meconium harbouring a microbial community [6–9]. However this remains a contentious area as it is difficult to completely rule out the possibility of external bacterial contamination. Shortly after birth, facultative anaerobic bacteria such as *Enterobacteriaceae* initially colonise the infant gastrointestinal tract, lowering redox potential and creating a suitable environment for the strict anaerobes who follow, mainly *Clostridium*, *Bacteroides* and *Bifidobacterium* [10]. The gut microbiota generally evolves from an immature and unstable state in infancy to a more complex, diverse and stable ecosystem by three years age and remaining so throughout adulthood [5, 11].

Several factors influence the composition and diversity of the neonatal intestinal microbiota including mode of delivery (vaginal vs. caesarean), feeding (breast vs. formula), hospital and home environment and antibiotic administration [12–15]. In addition to environmental factors, host genetics are also thought to play an important role in shaping the microbiota [16, 17].

Studies in monozygotic and dizygotic twins investigating the relative roles of host genetics and environmental effects in shaping gut microbiota composition have produced conflicting results. Many early twin studies report significantly higher similarity in related individuals compared with unrelated and with monozygotic twin pairs compared with dizygotic twins [18, 19]. In contrast, Turnbaugh et al showed that while genetically related individuals tend to share more of their gut microbiota than unrelated individuals, monozygotic twins were not significantly more similar than dizygotic twins[16, 20].

In this study, the gut microbiota in three dichorionic triplet sets were investigated. Dichorionic triplet sets contain a pair of monozygotic twins and a fraternal sibling. High-throughput sequencing was employed using 16S rRNA amplicons to compare the intestinal microbiota of the monozygotic pair to that of the fraternal triplet. The bifidobacterial population was also assessed using *rpoB* targeted pyrosequencing and species and strain diversity were analysed.

## Materials and Methods

### Participants and Sample Collection

Approval for this study was obtained from the Clinical Research Ethics Committee of the Cork Teaching Hospitals, Cork, Ireland. Informed written consent was obtained from the parents of each infant enrolled in the study. Three dichorionic triplet sets born by elective caesarean section at the Cork University Maternity Hospital were recruited (Table 1). Infants were excluded if they required oral antibiotics, required surgery, or had congenital abnormalities. Antibiotic administration occurred in two of the three triplet sets recruited, therefore, the healthy triplet set, set A, became the primary focus of this study. All infants were fed in the same manner; a mixture of expressed breast milk and formula (Table 1). Faecal samples were collected into a sterile container and stored at 4°C until delivery to laboratory; average processing time was within 12 hours of sampling. Samples were collected at 1, 2, 3 and 12 months of age in triplet set A and at 1 and 12 months in sets B and C.

**Table 1:** Clinical data from recruited triplet sets.

Triplet set	Infant-zygosity	Sex	Gestation (week + days)	Mode of delivery	Birth weight (g)	Mode of feeding	Hospital discharge (days)	Breast milk cessation (weeks)	Weaning commenced (weeks)	Antibiotic use	Antibiotic commenced (days)	Antibiotic duration (days)
A	1-MZ	F	34 + 2	ECS	1720	EBM + Formula	20	2	32	No	-	-
A	2-DZ	F	34 + 2	ECS	1890	EBM + Formula	20	3	32	No	-	-
A	3-MZ	F	34 + 2	ECS	1970	EBM + Formula	20	3	32	No	-	-
B	4-MZ	M	34 + 5	ECS	1990	EBM + Formula	18	3	22	No	-	-
B	5-MZ	M	34 + 5	ECS	2050	EBM + Formula	18	3	22	No	-	-
B	6-DZ	M	34 + 5	ECS	2160	EBM + Formula	18	3	22	Yes—GN—BP	<1 <1	<1 2
C	7-DZ	M	33 + 6	ECS	2100	EBM + Formula	23	3	18	Yes—GN—TP	24 25	7 6
C	8-MZ	M	33 + 6	ECS	2160	EBM + Formula	18	2	18	Yes—GN—FL	21 21	1 1
C	9-MZ	M	33 + 6	ECS	1720	EBM + Formula	16	2	18	No	-	-

MZ = Monozygotic; DZ = Dizygotic; ECS = Elective Caesarean Section; EBM = Expressed Breast Milk; Ab = Antibiotic; GN = Gentamicin; BP = Benzylpenicillin; TP = Teicoplanin; FL = Flucloxacillin

### 16S rRNA amplicon generation for 454 pyrosequencing

0.20g of fecal sample collected from triplet sets A, B and C at each time-point was transferred to a 2-mL screw-cap tube containing 0.25 g of a 1:1 mix of 0.1 mm and 1.5 mm diameter sterile zirconia beads plus a single 2.5mm diameter bead (BioSpec Products, Bartlesville, USA). Cells were mechanically disrupted using a Mini-Beadbeater-16 (BioSpec Products) for 2 mins at room temperature. DNA was purified using the QIAamp DNA Stool Mini Kit (Qiagen, Sussex, UK) according to manufacturer’s instructions. The microbiota composition of the samples was established by amplicon sequencing of the V4 region using universal 16S rRNA primers predicted to bind to 94.6% of all 16S rRNA genes as previously outlined by Claesson et al [21]. A forward primer 520f (5’-AYTGGGYDTAAAGNG) containing a distinct multiple identifier tag (MID) for each sample and a combination of four reverse primers R1 (5’-TACCRGGGTHTCTAAAGNG), R2 (TACCAGAGTATCTAATTC), R3 (5’-CTACDSRGGTMTCTAATC) and R4 (5’-TACNVGGGTATCTAATC) were utilised. PCR’s were performed under the following conditions: 94°C for 2 min followed by 35 cycles of 94°C for 1 min, 52°C for 1 min and 72°C for 1 min followed by 72°C for 2 min. PCR’s had a final volume of 50μl made up of 25 μl of Biomix Red (Bioline,Medical Supply Company, Dublin, Ireland), 1 μl forward primer (final concentration 0.15μM), 1 μl reverse primer (0.15μM), template DNA and sterile PCR grade water (Bioline). A negative control was included for each forward primer with a distinct MID, with template DNA being replaced with PCR-grade water. The Agencourt AMPure XP system (Beckman Coulter, Labplan, Co Kildare, Ireland) was used to clean the amplicons before quantification with the Quant-It Picogreen quantification kit (Bio-Sciences, Dublin, Ireland) and pooling for sequencing on a 454 Genome Sequencer FLX platform (Roche Diagnostics, West Sussex, UK) at the Teagasc Moorepark high throughput sequencing centre. DNA sequence reads from this study are available from the Sequence Read Archive (accession number PRJEB8333).

### *rpoB* amplicon generation for 454 pyrosequencing

DNA was purified from month 1 and month 12 stool samples from triplet sets A, B and C as outlined above. The highly conserved nature of the 16S V4 region makes it unsuitable for differentiating species of *Bifidobacterium*. A set of primers amplifying a 351bp region of the RNA polymerase β-subunit (*rpoB*) gene which have previously been successfully used for the differentiation of species of *Bifidobacterium* were utilised [15, 22]. Samples were amplified under the following conditions: 94°C for 2 min followed by 35 cycles of 94°C for 1 min, 60°C for 1 min and 72°C for 1 min followed by 72°C for 2 min. Subsequent steps were completed as outlined above for V4 amplicons.

### Bioinformatic analysis

Raw sequence reads were quality trimmed using the QIIME suite of tools version 1.8.0 [23]. Raw 16S rRNA reads failing to reach the quality criteria of a minimum quality score of 25, of a sequence length shorter than 200bps or not exact matches to barcode tags and primer sequences were discarded. Denoising, chimera detection and operational taxonomic unit (OTU) grouping at 97% similarity were performed in QIIME using USEARCH v7 [24]. Taxonomic ranks were assigned by alignment of OTUs using PyNAST [25]to the SILVA SSURef database release 111 [26]. For all OTU-based analyses, the original OTU table was rarefied to depths of 1,000 bacterial sequences per sample, to minimize the effects of read number differences between samples. In the *Bifidobacterium* analysis, the raw *rpoB* reads were quality trimmed using a locally installed version of the Ribosomal Database Project (RDP) Pyrosequencing Pipeline with read-lengths above 300 bp being used. Trimmed FASTA sequences were BLASTed against the NCBI non-redundant database using default parameters[27]. The resulting BLAST output was parsed through MEGAN using default parameters to extract phylum, family and genus counts[28]. In both cases alpha diversity indices were generated in QIIME and beta diversities were calculated based on unweighted UniFrac matrices [29]. Principal coordinate analysis (PCoA) were visualised using EMPeror v0.9.3-dev [30].

### Isolation, enumeration of *Bifidobacterium* sp. and DNA isolation

One gram of each faecal sample was mixed with 9ml maximum recovery diluent (Oxoid, Fisher Scientific, Dublin, Ireland) in a stomacher bag (Seward, VWR, Dublin, Ireland). Serial dilutions and plating were performed in a Whitley A85 anaerobic workstation (DW Scientific, Shipley, United Kingdom). For selective growth of bifidobacteria, 100 μl of dilutions were spread-plated onto de Man, Rogosa, Sharpe agar (MRS; Difco, Becton-Dickinson Ltd, Dublin, Ireland) supplemented with 0.05% (w/v) L-cysteine hydrochloride (Sigma-Aldrich, Dublin, Ireland), 100 μg ml^-1^ mupirocin (Oxoid) and 50U nystatin (Sigma Aldrich). Agar plates were incubated in anaerobic jars with AnaerocultA gas packs (Merck Millipore Ltd, Cork, Ireland) at 37°C for 72 hours. Bacterial counts were recorded as colony forming units (CFU) per gram of faeces. Fifteen colonies from each sample were randomly selected to analyse the dominant *Bifidobacterium* population and subcultured in MRS agar supplemented with 0.05% L-cysteine hydrochloride and MRS broth for 24 to 48 hours. A bank of 360 putative *Bifidobacterium* isolates was generated and maintained at –80°C in 40% glycerol (Sigma-Aldrich). DNA was extracted from each isolate using the GenElute Bacterial Genomic DNA kit (Sigma-Aldrich) and stored at -20°C.

### 16S rRNA-internally transcribed spacer (ITS) sequence analysis

The identity of each putative *Bifidobacterium* isolate was confirmed by 16S rRNA-ITS sequence analysis. A 1.5-kb 16S rRNA gene-internally transcribed spacer (ITS) fragment was generated using a previously described method [31]. DNA sequencing of both strands was carried out by Beckman Coulter Genomics (Essex, UK) and strains were assigned to a particular species following comparison of the 16S rRNA-ITS sequences using the NCBI BLAST database (http://www.ncbi.nlm.nih.gov/BLAST/).

### Pulsed Field Gel Electrophoresis

Genomic DNA was isolated from overnight cultures, lysed and digested with the restriction enzyme *Xba*I (New England Biolabs, Hitchin, UK) using a previously described method [32]. Electrophoresis was performed using a contour-clamped homogeneous electric field CHEF-DR III pulsed field system (Bio-Rad Laboratories, Hertfordshire, UK). Fragments were resolved with a linear ramp pulse time of 1- to 15-s for 18 h at 6 V/cm in a running buffer of 0.5X Tris-Boric Acid-EDTA maintained at a temperature of 14°C. A low-range PFGE marker (New England Biolabs) was also included as a molecular-mass marker. Gels were stained in 0.5μg/ml of ethidium bromide (Sigma-Aldrich) for 30 min, washed with distilled water for 5 x 20 minutes and visualized using an AlphaImager 3400 imaging system (ProteinSimple, CA, USA). Macrorestriction patterns were compared using the BioNumerics software version 6.5 (Applied Maths, Belgium). Dendrograms were constructed using UPGMA cluster analysis based on the Dice coefficient with 1.5% band tolerance. A cut-off at 90% similarity of the Dice coefficient was used to indicate identical PFGE patterns.

## Results

### Composition of the gut microbiota in the healthy dichorionic triplet set

**Fig 1:**
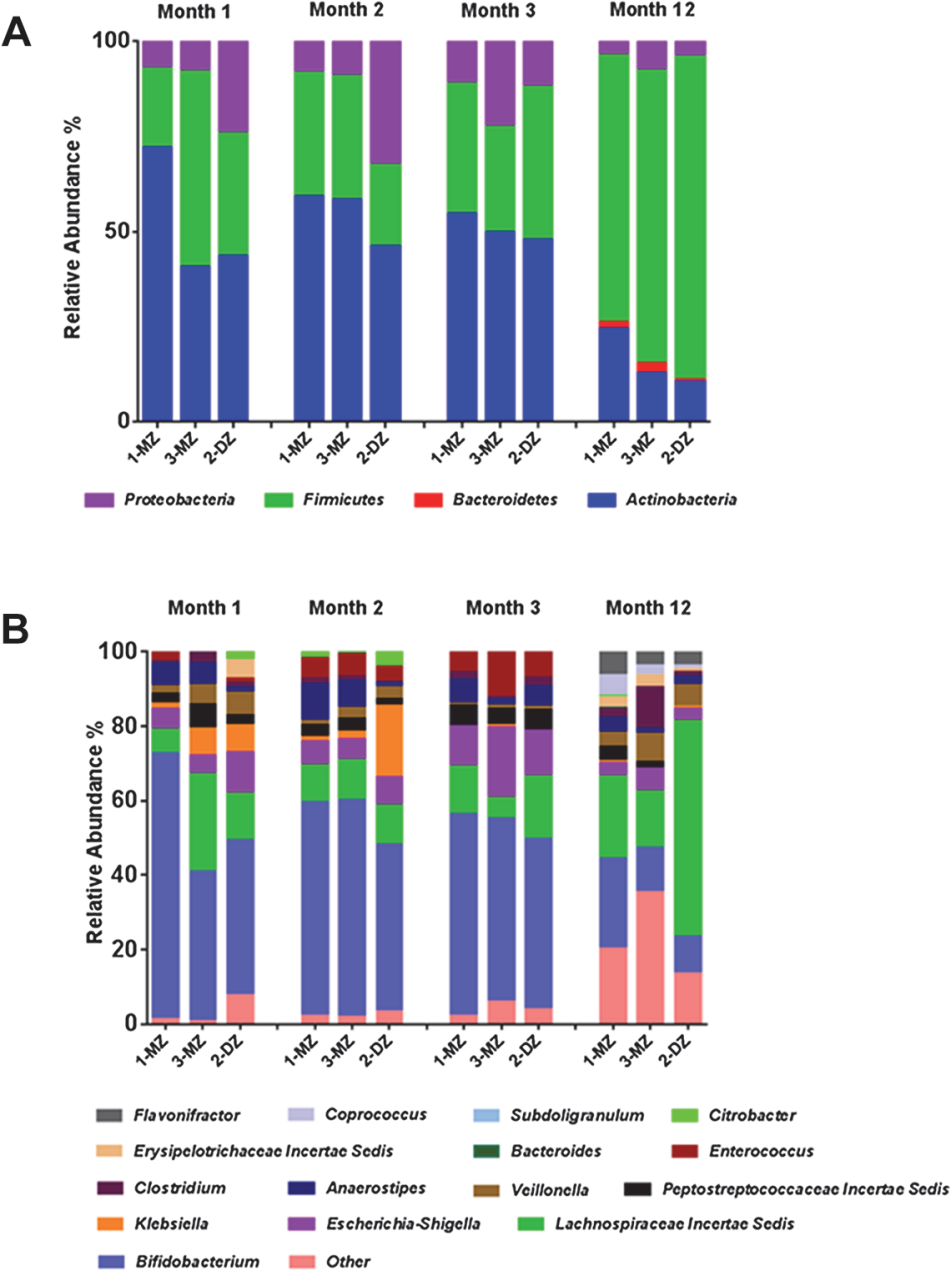
(A) Relative abundances of phylum level distributions of the fecal microbiota in triplet set A. (B)Relative abundances of genus level distributions of the fecal microbiota in triplet set A. The average relative abundance of phyla and genera in each infant was measured by the fraction of total 16S rRNA gene sequences. Each color represents a phylum/genus. Only major taxonomic groups are shown. MZ represents a monozygotic infant and DZ the dizygotic infant.

**Fig 2:**
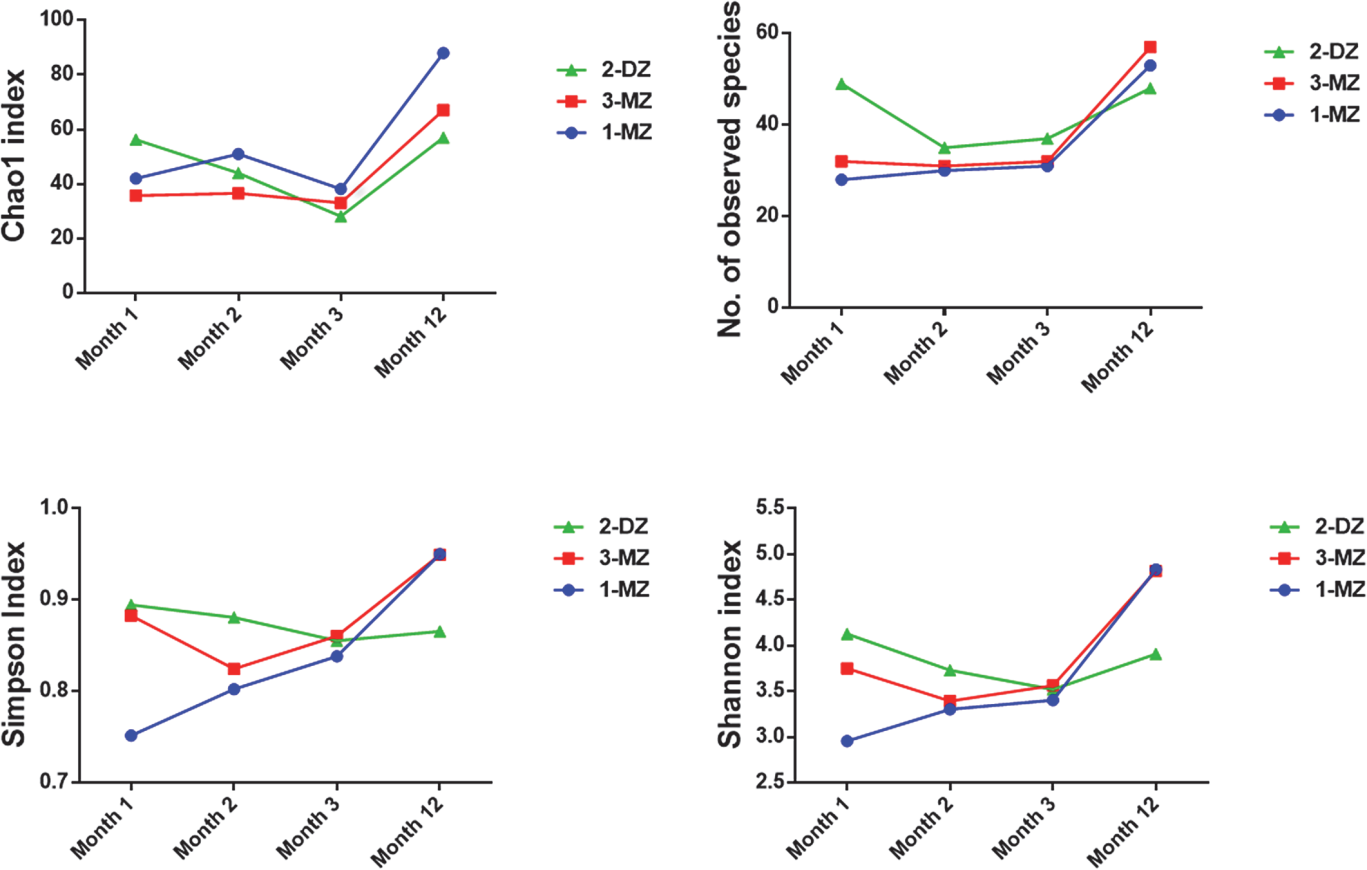
Comparison of diversity between samples from infants in triplet set A using different measures of alpha diversity. Alpha diversity indexes were calculated in QIIME from rarefied samples using the Chao1 index and number of observed species for richness, and the Shannon index and Simpson index for diversity and evenness.

454 pyrosequencing of V4 16S rRNA amplicons obtained from fecal samples from triplet set A at month 1, 2, 3 and 12 of life was performed. A total of 299,837 reads were generated, ranging from a minimum of 3,137 reads per sample to a maximum of 13,432 reads. Sequences were binned according to a 97% sequence identity cut-off and were assigned to 255 operational taxonomic units (OTU’s). Due to the inter-sample variation in read number, the OTU table was rarefied to 1000 reads, to facilitate comparison between samples. Analysis of the intestinal microbiota at month 1 revealed the presence of three phyla; Actinobacteria, Firmicutes and Proteobacteria. Sequences from Actinobacteria were predominant, with *Bifidobacterium* detected at a relative abundance of 71%% in 1-MZ, 40% in 3-MZ and 43% in the fraternal sibling, 2-DZ (Fig 1). Bacterial richness and diversity, as estimated by the Chao 1 metric and the Shannon index respectively, were observed to be highest in 2-DZ (Fig 2.). Bacterial diversity, measured by the Simpson and the Shannon index, was also highest in 2-DZ. By month 2, *Bifidobacterium* abundance was similar in all three infants; 58% for the monozygotic pair and 45% in 2-DZ (Fig 1). Members of *Enterobacteriaceae*, such as *Citrobacter* and *Klebsiella* were detected at a higher abundance in the fraternal triplet (Fig 1). At month 3, *Bifidobacterium* were detected at 54% and 49% in the monozygotic pair and 46% in 2-DZ, and overall bacterial richness and diversity were also comparable in all infants (Figs 1 and 2). An increase in diversity and richness from month 3 to month 12 was observed in all three infants. Phylum Firmicutes was found to predominate and Bacteroidetes were detected in all three infants (Fig 1). At genus level *Bifidobacterium* abundance had decreased in all infants and genera of the *Lachnospiraceae* family increased in all infants and were the dominant detectable family in the fraternal triplet, 2-MZ (Fig 1).

**Fig 3:**
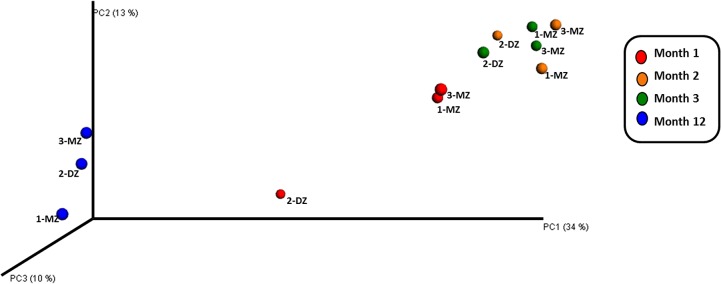
Unweighted UniFrac principal coordinates analysis (PCoA) plot. Unweighted UniFrac PCoA plot derived from 454 sequencing of V4 rRNA sequences from triplet set A fecal samples at month 1, 2, 3 and 12 comparing the presence/absence of operational taxonomic units (OTUs) and their phylogenetic relatedness.

Fig 3 represents principal coordinate analysis of the microbiota composition, based on unweighted UniFrac distances of the 16S rRNA sequences. Analysis at month 1 revealed a clear clustering of the monozygotic pair, 1-MZ and 3-MZ and a separation of the fraternal sibling, 2-DZ (Fig 3). However at months 2 and 3, while the monozygotic pair are still clustered closest together, the phylogenetic distance between the pair and the fraternal sibling was greatly reduced. By month 12, the separation of the fraternal infant from the monozygotic infants is no longer observed.

### Specific assessment of the bifidobacterial population in the healthy dichorionic triplet set

#### 454 Pyrosequencing of *rpoB* amplicons

**Fig 4:**
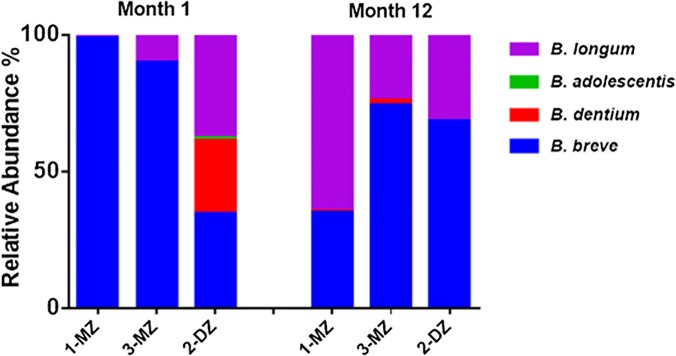
Relative abundances of bifidobacteria detected using *rpoB* amplicons in the healthy triplet set. MZ = monozygotic infant, DZ = dizygotic infant.

454 pyrosequencing was performed on *rpoB* amplicons obtained from month 1 and 12 fecal samples from triplet set A. While the 16S rRNA data presented above provides information on the relative abundances of the bifidobacteria present in the gut microbiota of the triplets, the *rpoB* data provides more detailed insights. High-throughput sequencing was again utilised but in this instance focused on the sequencing of amplicons corresponding to a region of the *Bifidobacterium* sp. RNA polymerase β-subunit gene, *rpoB*. The total number of reads obtained was 396,362 ranging from a minimum of 12,480 reads per sample to a maximum of 29,433 reads. This analysis revealed that the monozygotic pair were almost entirely dominated by *B*. *breve* at month 1 (Fig 4). In contrast, the fraternal infant, 2-DZ, exhibited greater diversity with *B*. *longum*, *B*. *adolescentis* and *B*. *dentium* species detected in addition to *B*. *breve*. In the case of *B*. *longum* it should be noted that the *rpoB* primers utilised are unable to distinguish between *B*. *longum* subspecies *longum* and *infantis*. By month 12 the species composition is similar across all three infants. Diversity has increased in the monozygotic pair and *B*. *breve* and *B*. *longum* were the predominant species in all three infants.

#### Enumeration and identification of culturable bifidobacterial species

**Fig 5:**
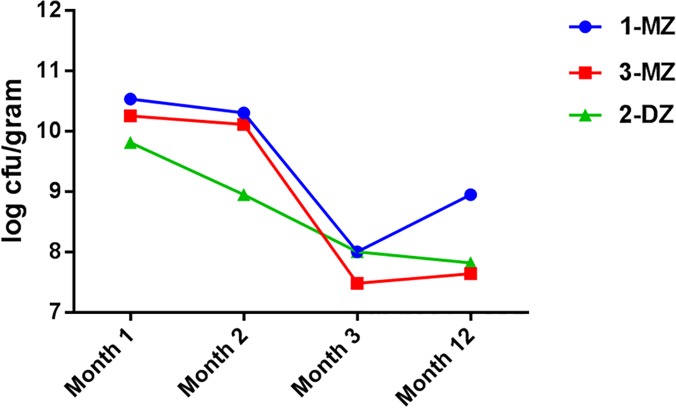
Levels of *Bifidobacterium* spp. triplet set A faecal samples. *Bifidobacterium* spp. were enumerated, log cfu/gram faeces, from faecal samples collected from infants in triplet set A at month 1, 2, 3 and 12. MZ = monozygotic infant, DZ = dizygotic infant.

Culturable bifidobacteria were detected in each infant at every sampling time point using mupirocin as a selective agent on solid media. The bifidobacterial counts at month 1 were highest for the monozygotic pair, 1-MZ and 3-MZ, at 10.53 and 10.25 log CFU/g faeces, respectively, compared with 2-DZ, at 9.81 log CFU/g faeces (Fig 5). At month 2 the highest bifidobacterial counts were detected in the monozygotic pair, 10.11 and 10.30 log CFU/g faeces compared to 8.95 log CFU/g faeces in 2-DZ. Culturable bifidobacteria numbers at month 3 were highest in 1-MZ and 2-DZ, 8.0 log CFU/g faeces in both, versus 7.48 log CFU/g faeces in 3-MZ. At month 12, the bifidobacterial counts had fallen in the fraternal triplet to 7.82 log CFU/g faeces and increased in 1-MZ and 3-MZ to 8.95 and 7.64 log CFU/g faeces respectively. BLAST results of each 16S rRNA gene – ITS sequence allowed species assignment of all isolates to four phylogenetic taxa, representing *B*. *breve*, *B*. *longum*, *B*. *dentium*, and *B*. *adolescentis*, correlating well with the *rpoB* data above. No new bifidobacterial species were identified as all sequences obtained showed more than 98% sequence identity to their nearest GenBank entry. The 16S-ITS sequences generated in this study have been uploaded to GenBank under the accession numbers KP791904-KP791967.

#### PFGE strain discrimination of *B*. *breve* isolates

**Fig 6:**
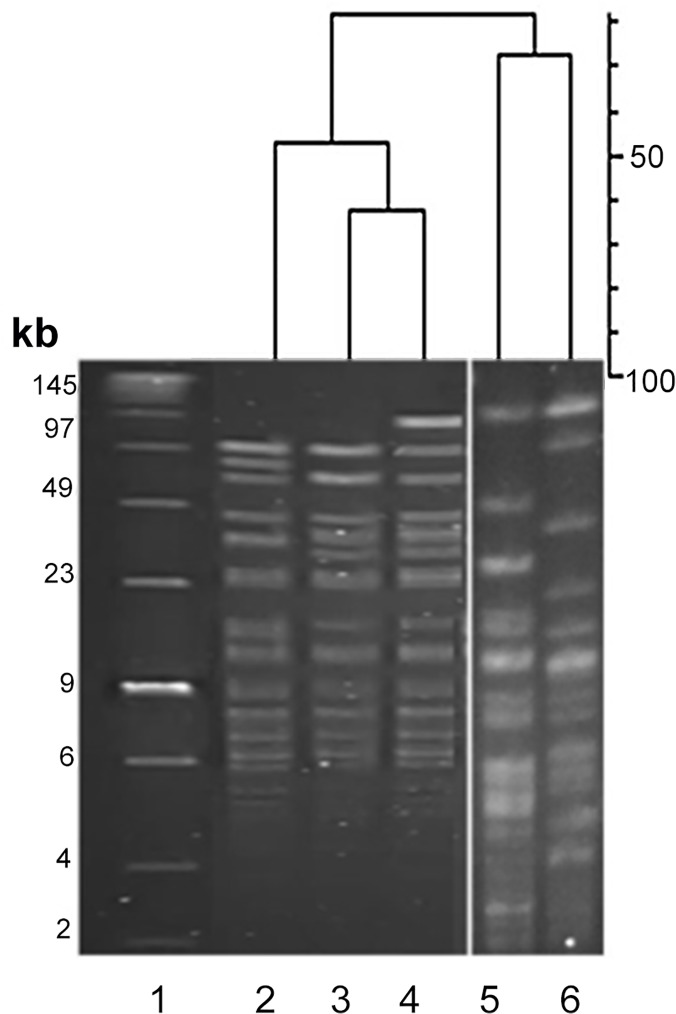
PFGE of *B*. *breve* isolates from triplet set A. PFGE macro-restriction patterns following genomic DNA digestion with the restriction enzyme XbaI of B. breve isolates. Lane 1 = Low molecular weight marker, Lane 2 = Pulsotype D, Lane 3 = Pulsotype A, Lane 4 = Pulsotype E, Lane 5 = Pulsotype B, Lane 6 = Pulsotype C.

PFGE following genomic digestion with the restriction enzyme *Xba*I has previously been shown to effectively discriminate between *Bifidobacterium* species [32]. The 80 *B*. *breve* isolates identified were resolved into 5 distinct pulsotypes (A–E) using the Dice coefficient at 90% similarity (Table 2 and Fig 6). Pulsotype A was the most dominant, accounting for 76% of isolates and persisted overtime, being detected at month 1 in all infants and at month 12 in 1-MZ and the fraternal sibling, 2-DZ. Pulsotypes D and E exhibited high similarity to A and differed by only 2 and 1 fragments respectively. These differences could be explained by a chromosomal rearrangement surrounding the restriction site in the case of D and the addition of a putative plasmid in E. Pulsotypes B and C were genetically distinct from others but transient, being detected only at month 1.

**Table 2:** Frequency of pulsotypes representing 80 *B*. *breve* faecal isolates from dichorionic triplet set.

Pulsotype	1-MZ	2-DZ	3-MZ	1-MZ	2-DZ	3-MZ
	Month 1			Month 12		
A	15	4	13	12	11	15
B		1				
C			2			
D				3		
E					4	

Values represent the number of isolates matching a particular pulsotype.

### Analysis of the antibiotic treated dichorionic triplet sets

In addition to the primary healthy triplet set, samples from the two antibiotic treated triplet sets were also subject to 454 pyrosequencing at month 1 and month 12.

#### Set B

**Fig 7:**
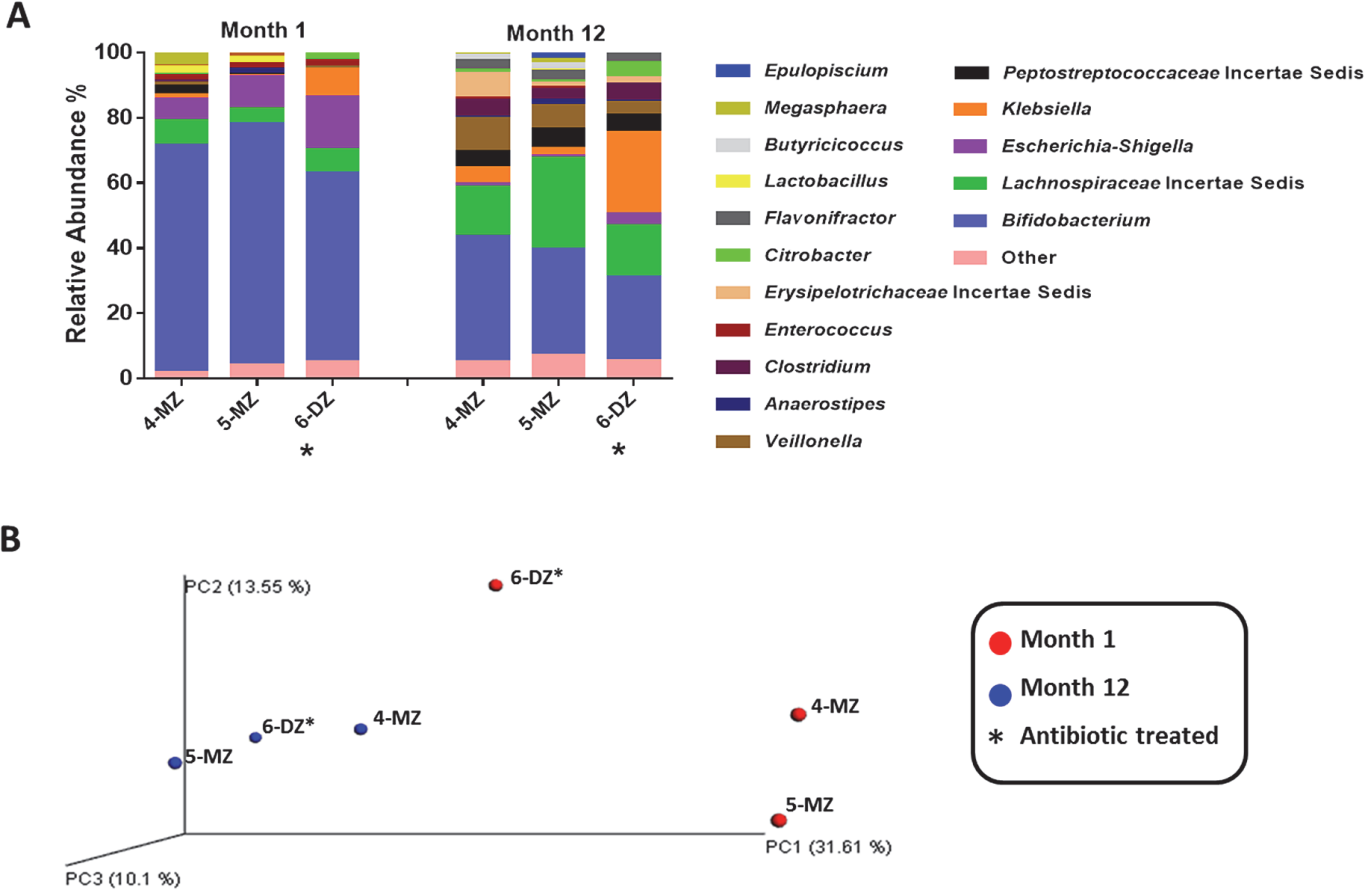
A) Relative abundances of genus level distributions of the fecal microbiota in the antibiotic treated triplet set B. MZ = monozygotic; DZ = dizygotic infant; an asterisks (*) next to a circle denotes the antibiotic treated infant. B) Unweighted UniFrac Principal coordinate analysis (PCoA) of V4 sequences from antibiotic treated triplet set B.

In this triplet set, only the fraternal sibling, 6-DZ, received antibiotic administration in the first 24 hours of life (Table 1). At a phylum level, higher levels of Proteobacteria were detected 6-DZ at month 1. Specifically, relative abundances of members of the *Enterobacteriaceae* family; *Citrobacter*, *Klebsiella* and *Escherichia- Shigella*, were higher (Fig 7A). The relative abundance of *Bifidobacterium* was lower in 6-DZ, 48%, compared with 65% and 61%. At month 12, *Bifidobacterium* abundances have decreased in all three infants and overall diversity has increased (Fig 7A). Principal coordinate analysis of the microbiota composition at month 1 is represented in Fig 7B. At month 1 the data points are quite disperse, though the monozygotic pair cluster more closely together than the antibiotic treated infant, 6-DZ. At month 12, the infants cluster tighter together and separation of the antibiotic treated infant is not observed.

**Fig 8:**
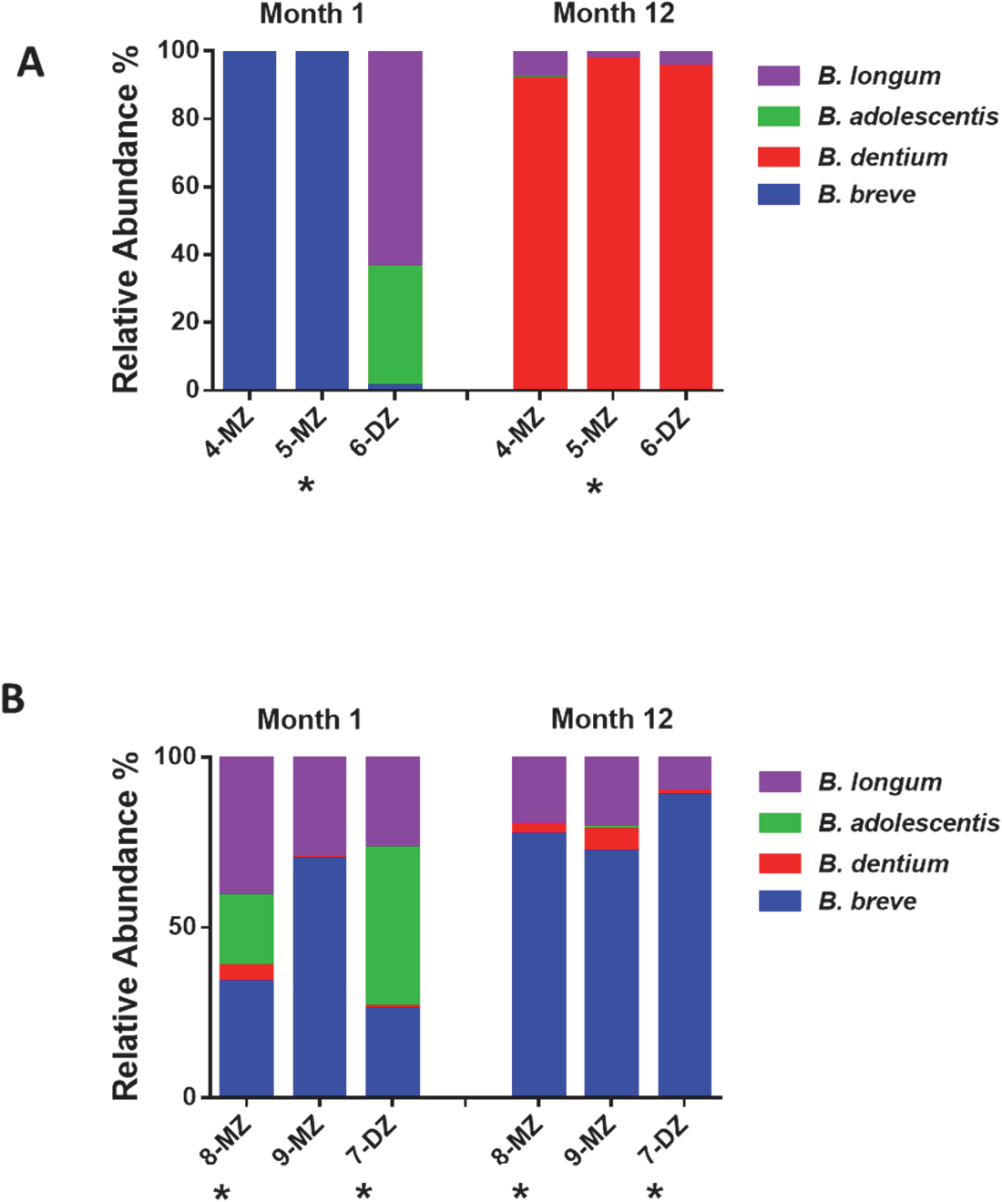
A) Relative abundances of bifidobacteria detected using *rpoB* amplicons for 454 pyrosequencing in the antibiotic treated triplet set B. MZ = monozygotic; DZ = dizygotic triplet; an asterisks (*) next to a circle denotes the antibiotic treated infant. B) Relative abundances of bifidobacteria detected using *rpoB* amplicons for 454 pyrosequencing in the antibiotic treated triplet set C. MZ = monozygotic; DZ = dizygotic triplet; an asterisks (*) next to a circle denotes the antibiotic treated infant.

*rpoB* amplicons were also pyrosequenced for this set and revealed that the monozygotic pair were comprised entirely of *B*. *breve* sequences at month 1 (Fig 8A). In contrast to its healthy siblings, *B*. *breve* represented only 1% of sequences in 6-DZ, while sequences representing *B*. *longum* and *B*. *adolescentis* predominated. At month 12, *B*. *dentium* represented > 90% of sequences for all three infants.

#### Set C

**Fig 9:**
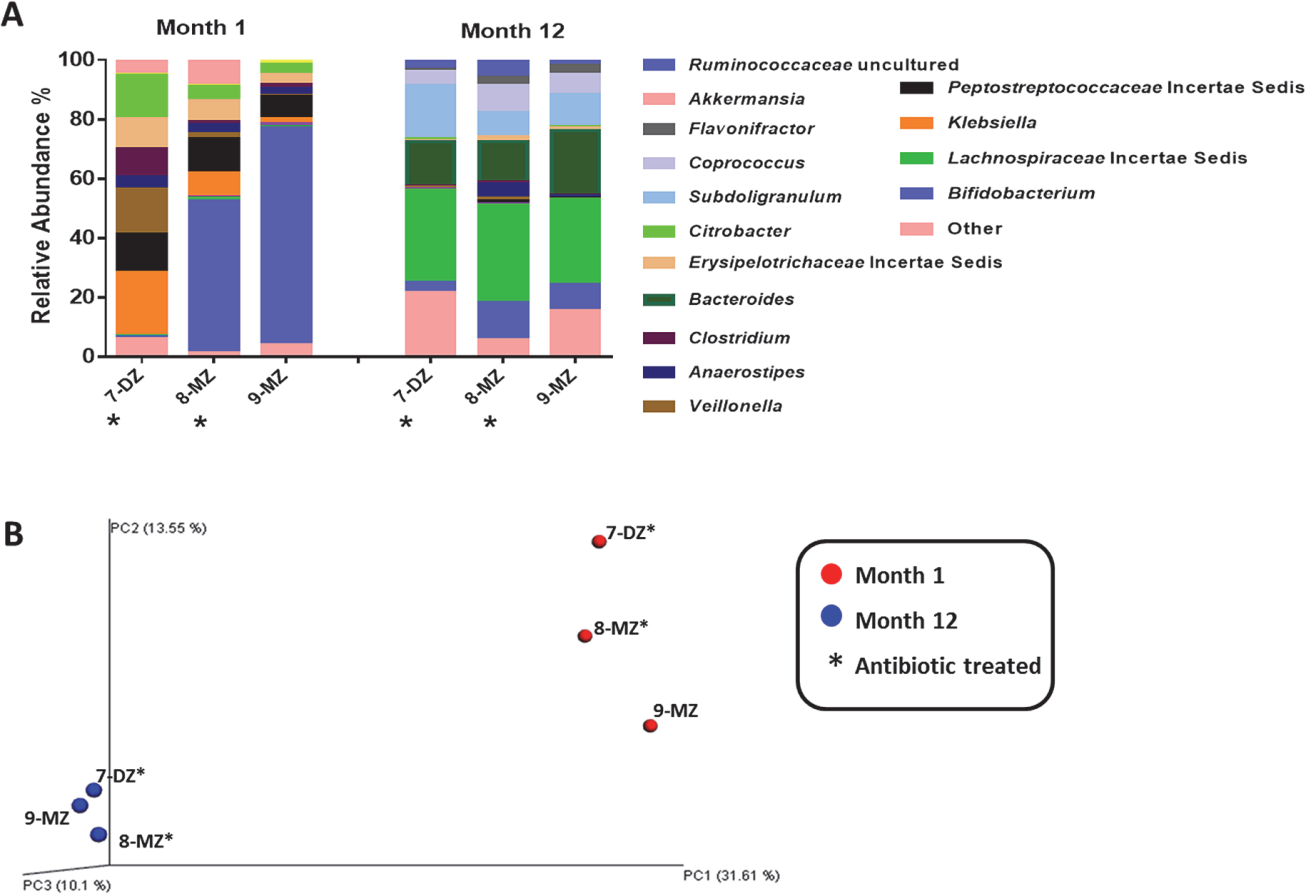
A) Relative abundances of bifidobacteria detected using rpoB amplicons for 454 pyrosequencing in the antibiotic treated triplet set B. MZ = monozygotic; DZ = dizygotic triplet; an asterisks (*) next to a circle denotes the antibiotic treated infant. B) Relative abundances of bifidobacteria detected using rpoB amplicons for 454 pyrosequencing in the antibiotic treated triplet set C. MZ = monozygotic; DZ = dizygotic triplet; an asterisks (*) next to a circle denotes the antibiotic treated infant.

In triplet set C, the fraternal sibling, 7-DZ, and one of the monozygotic pair, 8-MZ, received antibiotic administration at day 24 and 21, respectively (Table 1). Actinobacteria levels are dramatically reduced at month 1 in the treated infants; bifidobacteria represent 50% of sequences in 8-MZ compared to 73% in its healthy twin, 9-MZ. The most dramatic reduction is observed in the antibiotic treated fraternal sibling, 7-DZ, where *Bifidobacterium* constitute only 0.6% of the total relative abundance (Fig 9A). Relative abundances of members of the *Peptostreptococcaceae*, *Erysipelotrichaceae* and *Enterobacteriaceae* families are higher in both antibiotic treated infants compared with the healthy triplet. At month 12, the microbiota composition of all three infants are comparable, *Bifidobacterium* levels were low and the predominant species are members of the *Lachnospiraceae* family and the genera *Bacteroides* and *Subdoligranulum* (Fig 9A). At month 1, the data-point in the principal coordinate analysis corresponding to the antibiotic treated monozygotic infant, 8-MZ, is equidistant from its monozygotic twin, 9-MZ, and the antibiotic treated fraternal infant, 7-DZ (Fig 9B). At month 12 all three infants cluster closely together.

Analysis of the *rpoB* amplicons for this set revealed higher diversity in the antibiotic treated infants at month 1 with *B*. *breve*, *B*. *dentium*, *B*. *adolescentis* and *B*. *longum* species identified (Fig 8B). *B*. *longum* and *B*. *dentium* were also identified in the healthy infant however *B*. *breve* predominated. A similar profile was observed in all three infants at month 12, with *B*. *breve* the dominant species in all three.

## Discussion

To examine the roles of environmental versus host genetic factors in shaping gut microbiota composition, dichorionic triplet sets were investigated. Dichorionic triplets pose a unique informative study design as they contain monozygotic twins and a fraternal sibling with similar pre- and post-natal environmental factors, thus serving as an ideal control. To our knowledge this is the first time such an approach using multiple dichorionic triplet sets has been used to explore the effects of environment and genetics factors on microbiota development.

A limitation of the study is the small sample size; this is due to the difficulty in recruiting dichorionic triplet sets. The incidence of triplet births in 2012 at the Cork University Maternity Hospital was 13 sets (0.05% of total births), of which 5 were dichorionic triplets. Antibiotic administration is very common in these infants as they are typically preterm and caesarean section-delivered with an increased risk of infection. Two of the three triplet sets recruited received antibiotic administration within the first 24 hours of life. Therefore, the primary focus of this study became the healthy triplet set to provide a deeper insight into the effects of environmental and genetic factors on the development of the microbiota using pyrosequencing technology and culture dependent methods. For comparative purposes the microbiota of the antibiotic treated triplet sets was also assessed as an exploratory analysis.

In the healthy triplet set, pyrosequencing of the V4 region revealed greater similarity in the microbiota profile of the monozygotic pair compared with the fraternal sibling at month 1. By month 12 the profile was more uniform between the three infants. There is a transition from communities enriched with earlier colonisers such as *Bifidobacterium* to a more diverse microbiota. The strong clustering of the monozygotic pair at month 1 and the separation of the fraternal sibling as revealed by PCoA analysis is striking. At months 2 and 3 the phylogenetic distance between the monozygotic pair and the fraternal sibling has greatly reduced. By month 12 the microbiota composition has evolved further and the monozygotic pair no longer separate from the fraternal infant.

To specifically assess the bifidobacteria population, *Bifidobacterium* species diversity was analysed using *rpoB* targeted pyrosequencing. At month 1, the monozygotic pair were almost entirely dominated by *B*. *breve*, with greater species diversity detected in the fraternal sibling. At month 12, the bifidobacterial population was similar in all three. Analysis of the strain diversity of the culturable *B*. *breve* population by PFGE revealed a lack of diversity. This lack of strain diversity between the triplets is noteworthy. A recent study by our group investigated the bifidobacterial strain diversity in 51 unrelated infants and found a high level of diversity, with only one strain found in two different infants [33]. Here, the monozygotic pair were no more similar to each other than the fraternal sibling suggesting an environmental influence. Evidence for horizontal transmission in the environment has also been seen in a study of the *Clostridium perfringens* PFGE profiles from elderly subjects. Unrelated subjects from the same residential care location were reported to have identical strains [34].

Antibiotic exposure early in life, even short term, has been shown to significantly disrupt microbiota development in infants [15]. In both of the antibiotic treated triplet sets investigated here, decreased levels of Actinobacteria and increased *Enterobacteriaceae* were observed regardless of zygosity at month 1. By month 12, early antibiotic exposure appears to no longer exert such a strong influence on microbiota composition. The observation that the microbiota profile is similar in all infants at month 12 is of importance as a number of studies have suggested that exposure to antibiotics in early life may have long term effects on the microbiota composition. Indeed early antibiotic use has been associated with the development of allergic asthma, obesity and inflammatory bowel disease later in life [35–39]. A follow up with the triplet sets in this study later in life would be of benefit to investigate the impact of early antibiotic use on the development of disease states.

Specific analysis of the bifidobacterial population in these antibiotic treated infants revealed higher diversity and decreased levels of *B*. *breve* compared to their healthy siblings at month 1. In a previous study we investigated the effect of short-term antibiotic administration on the infant gut microbiota until 8 weeks of age and also found B. breve to be higher in control infants compared to the antibiotic treated cohort [15].At month12, the bifidobacterial composition at month 12 is similar in both healthy and antibiotic treated infants.

Other studies using dichorionic triplet sets to investigate shared factors in determining the gut microbiota include a publication by Stewart et al, which explored the development of the gut microbiome of preterm infants including that of a single dichorionic triplet set [40]. Here it was reported that the microbiota composition was comparable between all infants in the triplet set. It is important to note that each infant in that study received antibiotic administration within 24 hours of life and much like the antibiotic treated infants in our study it is likely that antibiotic administration was a major determinant of the community structure. Subramanian et al. sampled the gut microbiomes of healthy and malnourished infants in Bangladesh including a single dichorionic triplet set [41]. The maturity of the infants microbiota was measured and the monozygotic pair were not more correlated than their fraternal sibling. As in the case of Stewart et al, antibiotic use was also reported for these infants making the presence of a healthy dichorionic triplet set in our study of particular value.

Our observations require confirmation with future studies of dichorionic triplet sets but the data suggests that while initially host genetics play a major role in determining the microbial community composition, by year one environmental factors are the major determinant in healthy infants. In the case of early life antibiotic administration, this appears to be more of a determinant of the community composition at month 1 than any other factor including host genetics.
